# The Voltage-Gated Calcium Channel EGL-19 Acts on Glia to Drive Olfactory Adaptation

**DOI:** 10.3389/fnmol.2022.907064

**Published:** 2022-06-17

**Authors:** Du Chen, Hankui Cheng, Siyan Liu, Umar Al-Sheikh, Yuedan Fan, Duo Duan, Wenjuan Zou, Linhui Zhu, Lijun Kang

**Affiliations:** ^1^Department of Neurobiology and Department of Neurology of the Fourth Affiliated Hospital, Zhejiang University, School of Medicine, Hangzhou, China; ^2^NHC and CAMS Key Laboratory of Medical Neurobiology, MOE Frontier Science Center for Brain Research and Brain-Machine Integration, School of Brain Science and Brain Medicine, Zhejiang University, Hangzhou, China

**Keywords:** glia, glia-neuron interaction, olfaction, calcium channel, adaptation

## Abstract

Calcium channelopathies have been strongly linked to cardiovascular, muscular, neurological and psychiatric disorders. The voltage-gated calcium channels (VGCC) are vital transducers of membrane potential changes to facilitate the dynamics of calcium ions and release of neurotransmitter. Whether these channels function in the glial cell to mediate calcium variations and regulate behavioral outputs, is poorly understood. Our results showed that odorant and mechanical stimuli evoked robust calcium increases in the amphid sheath (AMsh) glia from *C. elegans*, which were largely dependent on the L-Type VGCC EGL-19. Moreover, EGL-19 modulates the morphologies of both ASH sensory neurons and AMsh glia. Tissue-specific knock-down of EGL-19 in AMsh glia regulated sensory adaptability of ASH neurons and promoted olfactory adaptation. Our results reveal a novel role of glial L-Type VGCC EGL-19 on olfaction, lead to improved understanding of the functions of VGCCs in sensory transduction.

## Introduction

Behaviors are strictly dependent on the appropriate flow of information within neuronal circuits consisting of both inhibitory and excitatory neurons. Voltage-gated calcium channels (VGCC) function in the cell membrane to mediate an influx of extracellular calcium in response to membrane depolarization (Catterall, 2000, 2011). In excitable cells, the entering calcium ions triggers neurotransmitter release from nerve terminals and muscle contraction through excitation-contraction coupling. In addition, calcium can act as second messengers to initiate longer lasting effects on gene expression and synaptic plasticity. Thus, VGCCs serve as an important link between electrical signaling and numerous important cellular processes in the nervous system (Catterall, 2000, 2011; Tam et al., 2000). VGCCs are mostly studied in excitable cells, like neurons and muscles; however, VGCCs have also been shown to play essential roles in non-excitable cells, including breast cancers and osteoclasts (Catterall, 2011; Pitt et al., 2021). Further glial cells are an indispensable component of the nervous system, but they are not easy to be excited electrically. Studies have revealed that glial cells exhibited spontaneous microdomain Ca^2+^ transients and also can be activated by odor and mechanical stimuli in a calcium-dependent manner, and function in olfactory detection, transduction and processing (Liu et al., 2014; Ding et al., 2015; Duan et al., 2020; Fernandez-Abascal et al., 2022). However, there are relatively few studies about glial calcium transients in regards to functions of VGCCs in glia cells.

Vertebrate VGCCs typically consist of the pore forming α1 subunit and the accessory subunits β, α2δ, and γ. The α1 subunit is a membrane spanning protein with the voltage sensor and a pore-forming structure in the center, which, when open, selectively allows Ca^2+^ flux across the membrane (Catterall, 2000, 2011). Vertebrate α1 subunits are classified into three major types: L-type high voltage activated (HVA), non-L-type HVA, and low voltage activated (LVA), or T-type channels (Catterall, 2011; Stout and Parpura, 2011; Pitt et al., 2021). The genetic model *Caenorhabditis elegans* possess all main types of VGCCs. *C. elegans* have five putative α1 subunits, encoded by *egl-19, cca-1, unc-2, nca-1*, and *nca-2*, and two α2δ subunits and two β subunits (Bargmann, 1998; Hobert, 2013). EGL-19, UNC-2, and CCA-1 are homologs to vertebrate α1 subunits conducting L-type, non-L-type, and LVA T-type channels, respectively (Bargmann, 1998; Jospin et al., 2002; Shtonda and Avery, 2005; Laine et al., 2011; Hobert, 2013). Equipped with 302 neurons and 56 glial cells, *C. elegans* is an good excellent model to study the function of VGCCs in glial cells.

EGL-19, a homolog of mammalian CACNA1 which is known for the rare human channelopathy Timothy syndrome type 1, carries L-type VGCC currents in *C. elegans* (Shtonda and Avery, 2005; Laine et al., 2011; Hobert, 2013; Lagoy et al., 2018). EGL-19 can not only affect the action potentials of neurons and muscles (Shtonda and Avery, 2005; Liu et al., 2011, 2018), but also affect calcium dynamics of ASH neurons and regulate their adaptability under sensory stimulation (Kato et al., 2014). Nevertheless, the mechanism by which EGL-19 regulates the adaptability of ASH neurons is unclear. Interestingly, our previous study revealed that the sheath glial cells of the amphid organ (AMsh glia) can also regulate the adaptability of ASH neurons (Duan et al., 2020), raising a question that whether EGL-19 affects the function of AMsh glial cells to regulate sensory adaptation.

Here, we found that EGL-19 modulated the morphologies of both neurons and glia. Odor- and touch- induced calcium signals in the AMsh glia were weakened in worms with the *egl-19* dysfunction mutation. We further revealed that AMsh glial EGL-19 contributes to sensory adaptability of ASH neurons and olfactory adaptation.

## Results

### The VGCC EGL-19 Is Essential for Olfactory Sensing of AMsh Glia

*Caenorhabditis elegans* respond with avoidance behaviors to a wide range of odorants in a dose-dependent manner to avoid tissue damages (Yoshida et al., 2012; Duan et al., 2020; Cheng et al., 2021). Our previous studies showed that aversive olfactory stimuli directly induced calcium variations in both olfactory neurons and AMsh glia (Duan et al., 2020; Cheng et al., 2021). It has been reported that the voltage-gated calcium channel EGL-19 is required for olfaction of ASH neurons (Duan et al., 2020). Therefore, we first checked whether voltage-gated calcium channels are involved in odorant induced responses in AMsh glia.

**Figure 1:**
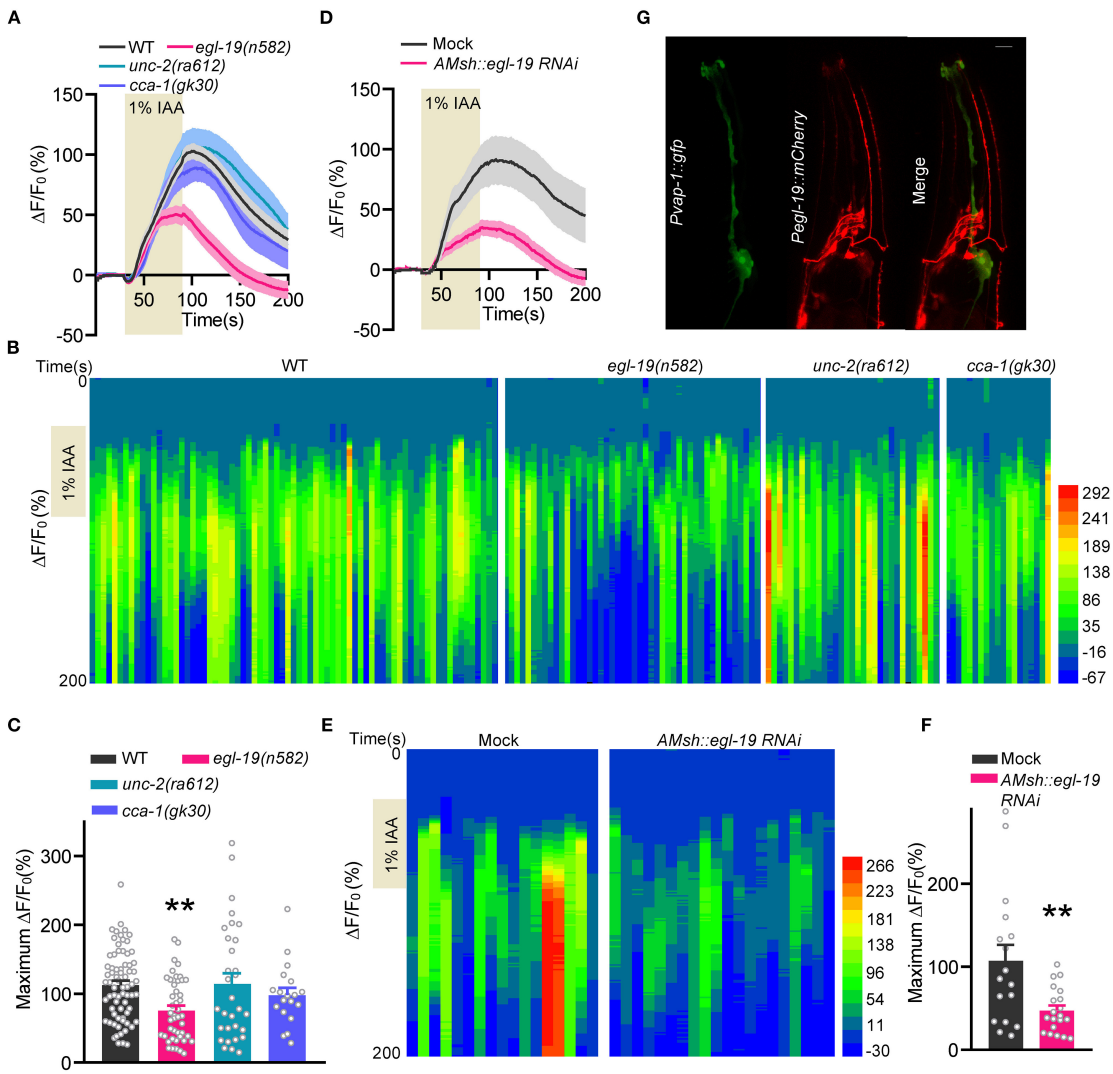
EGL-19 is involved in IAA-evoked Ca^2+^ increases in AMsh glia. **(A–C)** IAA (1/100)-evoked calcium increases of AMsh glia in wild type, *egl-19(n582)* mutants, *unc-2(ra612)* mutants, and *cca-1(gk30)* mutants. **(A)** Calcium responses. Note that the solid lines show the average fluorescence changes and the shading indicates SEM. **(B)** Heat map of calcium responses. **(C)** Maximum ΔF/F_0_ changes. IAA were dissolved 1:1 in DMSO as stock solution and then diluted in the bath solution to a final concentration of 1:100 before use. ΔF and F_0_ are defined as the fluorescence changes after IAA addition and the average fluorescence intensity from the last 10 s before IAA addition, respectively. Wild type (ST1071), *n* = 70; *egl-19(n582)* (ST2889), *n* = 45; *unc-2(ra612)* (ST2990), *n* = 31; *cca-1(gk30)* (ST2991)*, n* = 29; unpaired *t*-test. **(D–F)** Specific knockdown of *egl-19* in AMsh glia led to defective AMsh glial calcium increases in response to IAA (1/100). **(D)** Calcium responses. **(E)** Heat map of calcium responses. **(F)** Maximum ΔF/F_0_ changes. Mock (ST2674), *n* = 17; *AMsh egl-19 RNAi* (ST2729)*, n* = 20; unpaired *t*-test. **(G)** The co-localization of *Pegl-19::mCherry* with *Pvap-1::GFP* supports that EGL-19 is expressed in AMsh glia. Scale bar, 10 μm Error bars represent SEM. **p < 0.01.

We employed calcium imaging with transgenic worms expressing a genetically encoded calcium reporter GCaMP5.0 under the control of the AMsh glia-specific promoter *vap-1* (*Pvap-1::GCaMP5.0*) (Bacaj et al., 2008; Ding et al., 2015; Duan et al., 2020). Consistent with our previous report (Duan et al., 2020), AMsh glia displayed robust calcium increases in response to isoamyl alcohol (IAA, 1:100 in bath solution) (Figure 1A). Remarkably, IAA-evoked calcium increases were significantly reduced in *egl-19(n582)* mutants. Note that *egl-19(n582)* is a reduced-function allele, since knock-out alleles for *egl-19* are homozygous lethal (Stout and Parpura, 2011). Meanwhile, no defect was observed in mutations of either UNC-2 (P/Q-type α1 subunits) or CCA-1 (T-type α1 subunits) (Figures 1A–C). To better understand how EGL-19 affects the IAA-induced responses in AMsh glia, we conducted tissue-specific RNAi of *egl-19* in AMsh glia by transgenic microinjection (Duan et al., 2020). Remarkably, the AMsh glia-specific RNAi of *egl-19* largely reduced calcium transients of AMsh glia in response to IAA (Figures 1D–F). Collectively, these results support that EGL-19 VGCC is essential for olfaction of AMsh glia.

It has been implicated that EGL-19 is expressed in neurons and muscles (Lee et al., 1997; Jospin et al., 2002; Shtonda and Avery, 2005; Laine et al., 2011; Kato et al., 2014; Duan et al., 2020). However, no literature has reported whether EGL-19 is expressed in glial cells. We thus checked the co-localization of *Pegl-19::mCherry* and *Pvap-1::GFP*, and confirmed the expression of EGL-19 in AMsh glia (Figure 1G).

### Touch-Induced Calcium Increases in AMsh Glia Are Regulated by EGL-19

**Figure 2:**
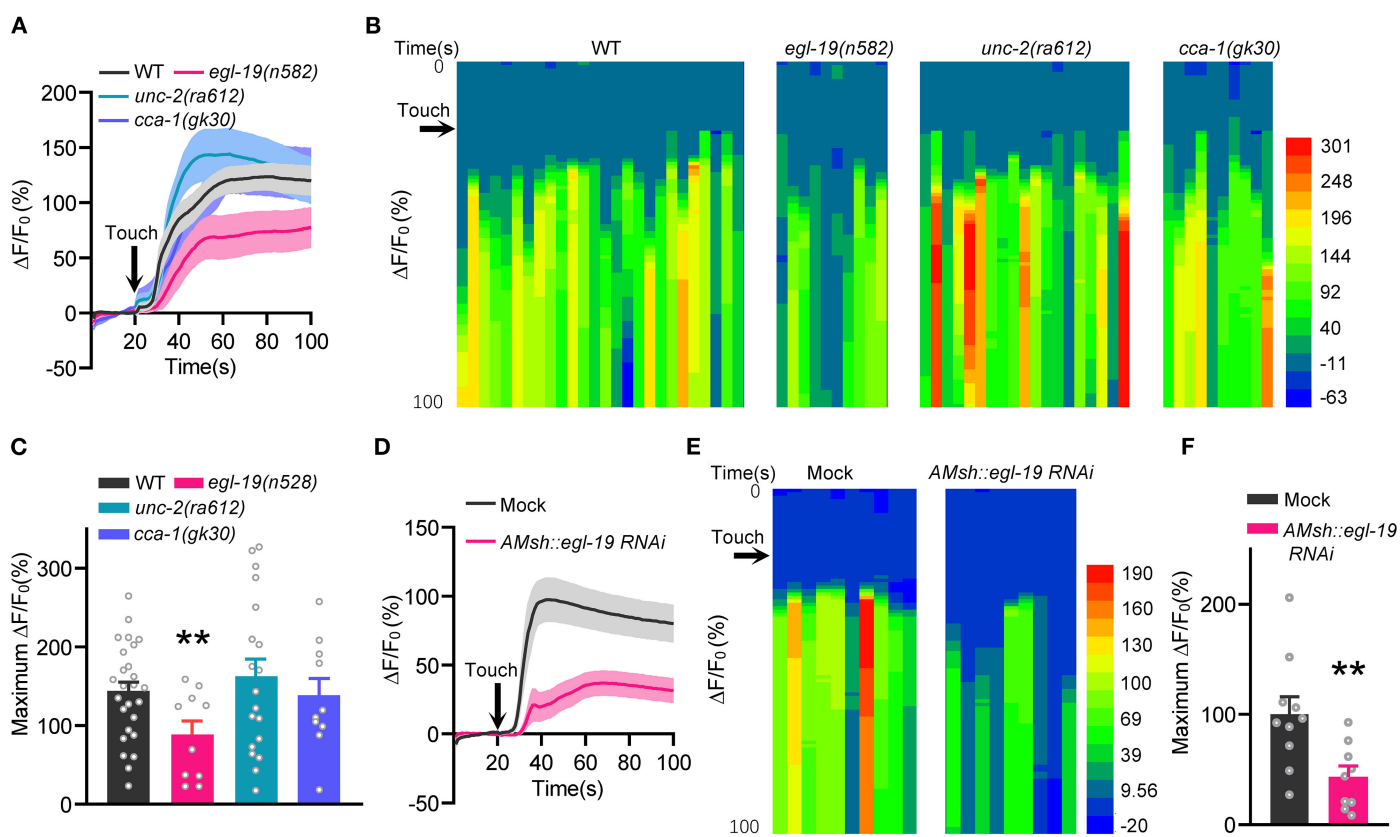
EGL-19 is essential for touch-evoked Ca^2+^ Increases in AMsh Glia. **(A–C)** Nose touch (2 Hz 20 μm displacement for 5 s) -evoked calcium variations of the AMsh glia in wild type, *egl-19(n582)* mutants, *unc-2(ra612)* mutants, and *cca-1(gk30)* mutants. **(A)** Calcium responses. **(B)** Heat map of calcium responses. **(C)** Maximum ΔF/F_0_ changes. Touch was performed toward the nose tip of the animals through a piezoelectric actuator, which can transform one volt pulse to 4.5 μm mechanical stimulation, wild type (ST1071), *n* = 26; *egl-19(n582)* (ST2889)*, n* = 10; *unc-2(ra612)* (ST2990)*, n* = 19; *cca-1(gk30)* (ST2991)*, n* = 10; unpaired *t*-test. **(D–F)** Specific knockdown of *egl-19* in AMsh glia led to defective AMsh glial calcium increases in response to nose touch. **(D)** Calcium responses. **(E)** Heat map of calcium responses. **(F)** Maximum ΔF/F_0_ changes. Mock (ST2674), *n* = 10; *AMsh egl-19 RNAi* (ST2729), *n* = 9; unpaired *t*-test. Error bars represent SEM. **p < 0.01.

AMsh glia are not only *bona fide* odorant receptor cells (Bianchi, 2020; Duan et al., 2020), but also mechano-receptor cells which can be activated by nose touch stimulation (Ding et al., 2015; Fernandez-Abascal et al., 2022). We next tested whether EGL-19 is required for touch-induced calcium increases in AMsh glia. Consistent with our previous report (Ding et al., 2015), robust calcium increases were observed in both the soma and the processes of AMsh glia under mechanical stimulation (stimuli with 20 μm displacement toward the nose tip of the worms, 2 Hz 5 s) (Figure 2A). Moreover, nose touch-induced calcium increases in AMsh glia were significantly reduced in *egl-19(n582)* mutants, but not in *unc-2(ra612)* or *cca-1(gk30)* mutants (Figures 2A–C). Further, the AMsh glia-specific RNAi of *egl-19* remarkably reduced nose touch-induced calcium increases in AMsh glia (Figures 2D–F). These results suggest that EGL-19 is also required for mechanical sensing of AMsh glia.

### Dysfunction of EGL-19 Causes Longer Soma and Processes in Both Neurons and Glia

**Figure 3:**
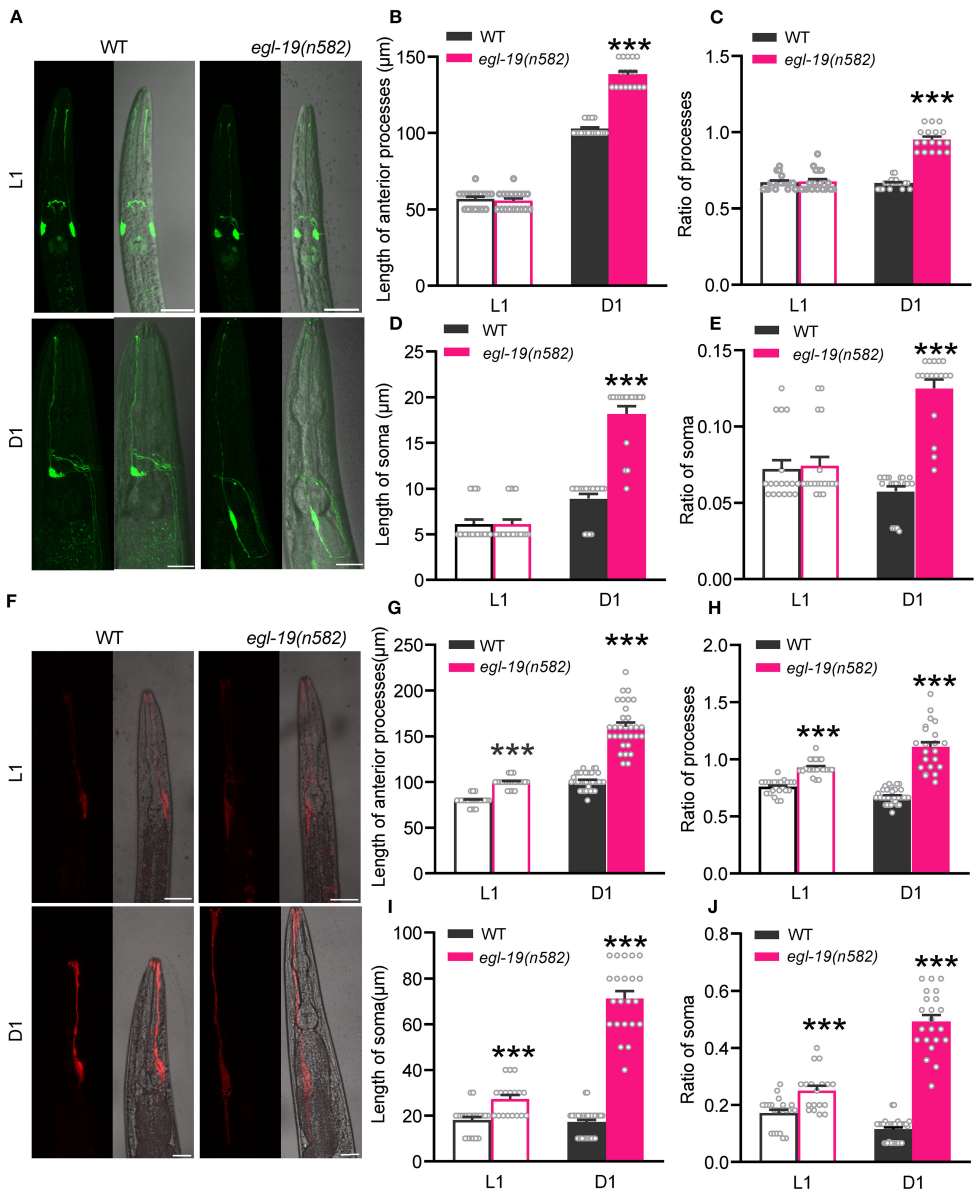
The morphologies of both AMsh glia and ASH neuron were modulated by EGL-19. **(A)** Representative morphologies of ASH neurons (green, labeled with *Psra-6::GFP*) in wild type and *egl-19(n582)* mutants at L1 stage (upper) and D1 stage (lower), respectively. Scale bar, 20 μm. **(B,D)** The lengths of the anterior processes **(B)** and soma **(D)** of ASH neurons in wild type and *egl-19(n582)* mutants at L1 and D1 stage. **(C,E)** The Ratio of the lengths of the anterior processes **(C)** and soma **(E)** of ASH neurons to the lengths of the second pharynx in wild type and *egl-19(n582)* mutants at L1 and D1 stage. The lengths of the second pharynx is calculated as the distance from nose tip to the middle of the second pharynx. L1 stage [WT (ST2169), *n* = 18; *egl-19(n582)* (ST2993), *n* = 18]; D1 stage [WT, *n* = 19; *egl-19(n582), n* = 17]; unpaired *t*-test. **(F)** Representative morphologies of AMsh glia (red, labeled with *Pvap-1::mCherry*) in wild type and *egl-19(n582)* mutants at L1 (upper) and D1 (lower), respectively. Scale bar, 20 μm. **(G,I)** The lengths of the anterior processes **(G)** and soma **(I)** of AMsh glia in wild type and *egl-19(n582)* mutants at L1 and D1 stage. **(H,J)** The Ratio of the lengths of the anterior processes **(H)** and soma **(J)** of AMsh glia to the lengths of the second pharynx in wild type and *egl-19(n582)* mutants at L1 and D1 stage. L1 stage [WT (ST826), *n* = 21; *egl-19(n582)* (ST993), *n* = 18]; D1 stage [WT, *n* = 21; *egl-19(n582), n* = 20]; unpaired *t*-test. Error bars represent SEM. ***p < 0.001.

A previous study reported that gain-of-function mutations of *egl-19* cause the growth of an ectopic process from the cell body of the ALM neurons (Buddell and Quinn, 2021). To better understand the phenotype of *egl-19* in olfaction, we labeled ASH neurons with GFP and checked the morphology by confocal microscopy. The morphology of ASH neurons in *egl-19(n582)* mutants at larva stage 1 (L1) was similar to that in wild type (Figure 3A). However, the soma of ASH neurons of *egl-19(n582)* mutants at adult day 1 (D1) stage were more posteriorly displaced, showing longer anterior processes [mean lengths of the anterior process of ASH neuron: 102.63 μm in wild type, and 138.24 μm in *egl-19(n582)*] (Figures 3B,C) and longer soma [mean lengths of the soma of ASH neurons: 8.95 and 18.18 μm in wild type and *egl-19(n582)* mutants, respectively] (Figures 3D,E). Moreover, we observed a much longer axon of ASH neurons that extended toward the posterior direction in *egl-19(n582)* animals.

Next we asked whether the morphology of AMsh glia is also affected by *egl-19* mutation. Both the soma and the processes were intact in *egl-19(n582)* mutants. Nevertheless, the AMsh glia showed longer anterior processes and longer soma in *egl-19(n582)* mutants than that in wild type animals both at larva stage and at adult (Figure 3F). The mean lengths of the anterior processes of AMsh glia were 79.57 and 99.52 μm in wild type and *egl-19(n582)* mutants, respectively, at larva stage 1 (L1), which turned to 100.97 μm in wild type and 160.65 μm in *egl-19(n582)* mutants at adult day 1 (D1). Meanwhile, the mean lengths of the soma of AMsh glia were 18.09 and 27.22 μm in wild type and *egl-19(n582)* mutants, respectively, at L1, and 17.14 μm in wild type and 71.36 μm in *egl-19(n582)* mutants at D1 (Figures 3G–J).

Collectively, these results suggest that EGL-19 VGCC modulates the morphologies of both neurons and glial cells.

We then asked whether EGL-19 functions in AMsh glia to modulate the morphologies of ASH neurons and AMsh glial cells. Interestingly, no significant morphological change of ASH neurons in the worms with the AMsh glia-specific RNAi of *egl-19* was observed. However, knocked down of EGL-19 in AMsh glia caused moderate morphological changes of AMsh glial cells with longer soma (Supplementary Figure 1). These results suggest that EGL-19 might modulate the morphology changes of ASH neurons in an AMsh glia-independent manner.

### EGL-19 Acts in AMsh Glia to Modulate Olfactory Adaptation

**Figure 4:**
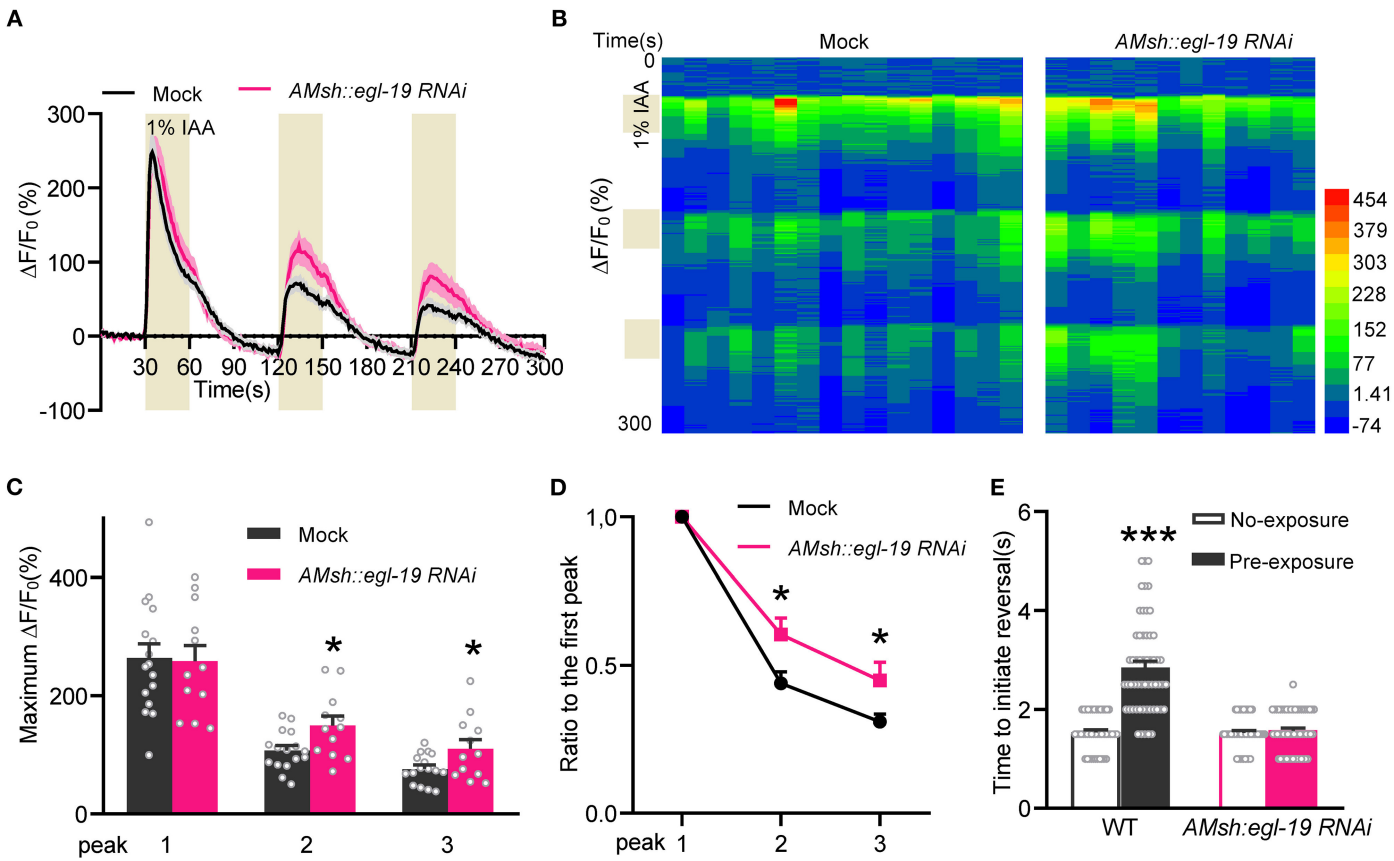
EGL-19 acts on AMsh glia to modulate olfactory adaptation. **(A–D)** The calcium response adaptation of ASH neurons upon repetitive IAA stimulation (1:100 IAA, 30 s-odorant pulses with 1-min intervals) was reduced in worms with AMsh glia-specific knockdown of *egl-19*. **(A)** Calcium responses. **(B)** Heat map. **(C)** Maximum ΔF/F_0_ changes of the ASH neuron in response to repetitive IAA. **(D)** The ratio of the average maximum ΔF/F_0_ changes of peak 2 and peak 3 to the maximum ΔF/F_0_ changes of the first peak. Mock (ST2996), *n* = 16; *AMsh egl-19 RNAi* (ST2995), *n* = 12; unpaired *t*-test. **(E)** Worms with AMsh glia-specific *egl-19* RNAi shown reduced behavioral adaptation to IAA. Five milliliter of undiluted odorant was dropped on the lid of a 3 cm-diameter NGM plate, then D1 adult worms were transferred into the plate and were covered by the odorant-containing lid. Sealed the plate with Parafilm. After 5-min adaptation, worms were washed twice with basal buffer (5 mM KPO_4_ [pH 6], 1 mM CaCl_2_, 1 mM MgSO_4_), and then were transferred onto normal NGM plates. Placed a 10 μL pipette tip filled with IAA in front of a forward-moving worm, and recorded the response latency for the worm to cease forward movement and initiate reversal (backward movement). No-exposure [Mock (ST2996), *n* = 46; *AMsh egl-19RNAi* (ST2995), *n* = 46]; pre-exposure [Mock (ST2996), *n* = 56; *AMsh egl-19RNAi* (ST2995), *n* = 57]; unpaired *t*-test. Error bars represent SEM. *p < 0.05; ***p < 0.001.

We next sought to characterize the function of EGL-19 in AMsh glia. Previously we reported that activation of AMsh glia under odorant stimulation suppresses ASH neurons *via* GABAergic signaling, which in turn promotes olfactory adaptation (Duan et al., 2020). So we tested the calcium response adaptation of ASH neurons upon three repetitive IAA stimuli with 1-min intervals. Consistent with our previous reports, ASH neurons adapted to repetitive IAA stimuli with decreased responses (Kato et al., 2014; Duan et al., 2020) (Figures 4A–D). Moreover, ASH neurons displayed reduced calcium response adaptation to repetitive IAA stimuli in the worms with the AMsh glia-specific RNAi of *egl-19*. These data suggest that EGL-19 is essential for the AMsh glia-regulated adaptability of ASH neurons.

ASH neurons play a dominant role in the olfactory avoidance behaviors (Bargmann et al., 1993; Hilliard et al., 2005; Kato et al., 2014; Duan et al., 2020). Since our observation showed that the adaptability of ASH neurons is regulated by AMsh glial EGL-19, we further established that the behavioral adaptation to pre-exposure IAA stimuli was largely reduced in the worms with the AMsh glia-specific RNAi of *egl-19* (Figure 4E), which supports that AMsh glial EGL-19 is essential for olfactory adaptation. Together, these results suggest that EGL-19 functions in the AMsh glia to promote olfactory adaptation by regulating the adaptability of ASH neurons.

## Discussion

The processing and storage of information by the nervous system requires the ability to modulate responses of excitable cells to stimulation. Adaptation occurs when prolonged or repetitive stimulation triggers processes that attenuate the response (Duan et al., 2020). It has been reported that L-type VGCC EGL-19 regulates the adaptability of ASH neurons under prolonged or repetitive stimulation but the mechanism is unclear (Kato et al., 2014). Neuronal VGCCs serve complex yet essential physiological functions *via* their pivotal roles in translating electrical signals into intracellular calcium elevations and associated downstream signaling pathways (Catterall, 2000, 2011). Several regulatory mechanisms have been implicated to act on VGCCs to indirectly regulate the amplitude, duration, adaptation and subcellular localization of the Ca^2+^ signal in neurons (Catterall, 2000, 2011). Compared to neurons, however, functional studies of VGCCs in glial cells have received less attention due to relatively weak electrical activities in these cells (Nedergaard and Verkhratsky, 2012).

In cultured cephalic sensilla sheath (CEPsh) glial cells respond to depolarization induced by high K^+^ solution with calcium increases mediated by VGCC genes *egl-19, cca-1* and *unc-2* (Stout and Parpura, 2011). However, the physiological meaning of VGCCs in glial cells is poorly understood. In this study, we found that EGL-19 L-type VGCC, but not UNC-2 P/Q-type or CCA-1 T-type VGCCs, is essential for calcium dynamics in AMsh glia in response to mechano- and odor- stimuli, correlating EGL-19 VGCC with the sensory function of AMsh glial cells. Moreover, we reveal that EGL-19 acts in AMsh glia to promote the adaptability of ASH neurons, which in turn modulating olfactory adaptation under odor stimulation.

Olfactory adaptation is a ubiquitously characteristic of olfaction from lower invertebrates to our humans, which prevents the saturation of transduction machinery and allows the retention of high sensitivity upon continuous or repetitive odorant stimulation (Colbert and Bargmann, 1995; Zufall and Leinders-Zufall, 2000). It has been implicated that most olfactory adaptation events occur cell-autonomously at the level of olfactory receptor neurons using distinct molecular mechanisms (Colbert and Bargmann, 1995; Zufall and Leinders-Zufall, 2000). In our previous study, we identified a novel mechanism by which the AMsh glia regulates olfactory adaptation by inhibiting the neighboring ASH neurons (Bianchi, 2020; Duan et al., 2020).

L-type VGCCs play a critical role in a wide spectrum of physiological processes, including neurotransmission, cell cycle, muscular contraction, cardiac action potential, and gene expression. The *egl-19* gene in *C. elegans* encodes the pore forming subunit for the L-type voltage gated calcium channel that is homologous to the *CACNA1C* gene in humans (Lagoy et al., 2018). Variants in *CACNA1C* are risk factors for autism and other psychiatric disorders such as schizophrenia (Strom et al., 2010; Lu et al., 2012; Li et al., 2015). Timothy syndrome is a syndromic form of autism that can be caused by either of three rare *de novo* mutations in *CACNA1C* (Bader et al., 2011). Meanwhile, a gain-of-function *egl-19* mutation, which is equivalent to the Timothy syndrome mutation in *CACNA1C*, causes defects in axon termination and neuronal polarity in the ALM neuron of *C. elegans* (Buddell and Quinn, 2021). Our results showed that the reduced-function mutation of *egl-19* results in longer processes and longer soma in both glia and neurons. It would be interesting to identify the relationship of glial VGCCs and olfaction in mammals and the psychiatric disorders including of autism and schizophrenia in future studies.

In this study, We found that EGL-19 function in both activity and morphology of AMsh glia cells and ASH neurons. Also, EGL-19 has an impact on ASH neuronal activity under repeated stimuli. AMsh glia suppress ASH neurons activity through GABA and mediate ASH olfactory adaptation (Duan et al., 2020). Interestingly, we found that ASH neuronal activity can be modulated by knocked down of EGL-19 in AMsh glial cells. Moreover, there was no significant morphological change of ASH neurons in the worms with the AMsh glia-specific RNAi of *egl-19*, even though moderate morphological changes were observed in AMsh glial cells. These results suggest that EGL-19 might cause the functional and morphological changes of ASH neurons though different pathways.

Taken together, our findings provide evidence that the EGL-19 L-type voltage gated calcium channel acts on the glia to modulate responses to environmental sensory cues and regulate olfactory adaptation. Our study has provided an improved understanding of the functions of VGCC in sensory transduction in both vertebrates and invertebrates, as well as the potential treatments of VGCC dysfunction-related hereditary diseases in humans.

## Methods

### Strains and Media

All nematode strains were cultivated at 20°C on nematode growth medium (NGM) plates seeded with the OP50 strain of *Escherichia coli* using standard methods previously described (Brenner, 1974). Well-fed Day 1 adult worms were used in all experiments expect measure of the morphologies of AMsh glia and ASH neurons. The strains used in this study are listed in the Table 1. Wild-type N2, *egl-19(n582), unc-2(ra612)*, and *cca-1(gk30)* mutant strains were all provided by the *Caenorhabditis* Genetic Center (CGC). *egl-19(n582)* used in this study is a L-type voltage-gated calcium channel (VGCC) α1 subunit reduced-function allele, because knock-out alleles for *egl-19* are homozygous lethal.

**Table 1:** Key resources table.

**Reagent or resource**	**Source**	**Identifier**
**Chemicals, peptides**, **and recombinant** **proteins**		
Isoamyl alcohol	Sigma-Aldrich	Cat#W205702
Ethyl alcohol	Sigma-Aldrich	Cat#459836
1-octanol	Sigma-Aldrich	Cat#V900239
**Critical commercial assays**		
In-Fusion HD Cloning Kit	Takara	Cat#639649
**Experimental models: organisms/strains**		
N_2_	CGC	ST348
*kanIs3[Pvap-1::mCherry+Pvap-1::GCaMP5.0+Punc-122::GFP]*	This study	ST1071
*kanIs8[Psra-6::mCherry+Psra-6::GCaMP5.0+Plin-44::GFP]*	This study	ST2169
*egl-19(n582);kanIs3[Pvap-1::mCherry+ Pvap-1::GCaMP5.0+Punc-122::GFP]*	This study	ST2889
*unc-2(ra612);kanIs3[Pvap-1::mCherry+ Pvap-1::GCaMP5.0+Punc-122::GFP]*	This study	ST2990
*cca-1(gk30);kanIs3[Pvap-1::mCherry+ Pvap-1::GCaMP5.0+Punc-122::GFP]*	This study	ST2991
*kanIs3[Pvap-1::mCherry+Pvap-1::GCaMP5.0+Punc-122::GFP];kanEx776[Pvap-1::egl-19::sense+Punc-122::mCherry]*	This study	ST2674
*kanIs3[Pvap-1::mCherry+Pvap-1::GCaMP5.0+Punc-122::GFP];kanEx858[Pvap-1::egl-19::sense+Pvap-1::egl-19::antisense+ Punc-122::mCherry]*	This study	ST2729
*kanEx859[Pegl-19::RFP+Pvap-1::GFP]*	This study	ST2732
*kanEx829[Pvap-1::mCherry+Pvap-1::GCaMP5.0+Punc-122::GFP]*	This study	ST826
*egl-19(n582);kanEx829[Pvap-1::mCherry+ Pvap-1::GCaMP5.0+Punc-122::GFP]*	This study	ST993
*egl-19(n582);kanIs8[Psra-6::mCherry+Psra-6::GCaMP5.0+Plin44::GFP]*	This study	ST2993
*kanIs8[Psra-6::mCherry+Psra-6::GCaMP5.0+Plin-44::GFP];KanEx861[Pvap-1::egl-19::sense+Pvap-1::egl-19::antisense+ Punc-122::mCherry]*	This study	ST2995
*kanIs8[Psra-6::mCherry+Psra-6::GCaMP5.0+Plin-44::GFP];KanEx861[Pvap-1::egl-19::sense+ Punc-122::mCherry]*	This study	ST2996
**Software and algorithms**		
ImageJ	NIH	https://imagej.nih.gov/ij/; RRID:SCR_003070
Micro-Manager	Vale Lab, UCSF	http://micro-manager.org, RRID:SCR_000415.

### Molecular Biology

Promoters were PCR-amplified from N_2_ genomic DNA and then recombined with specific donor vector fragments using the In-Fusion PCR Cloning Kit (TaKaRa Inc.). The following promoter fragments constructed with GFP or mCherry were used in this study: *Psra-6::GFP* (ASH neuron) and *Pvap-1::mCherry* (AMsh glia), which were used to identify ASH neurons and AMsh glia, respectively. *Psra-6::GCaMP5.0* and *Pvap-1::GCaMP5.0* were used for calcium imaging in ASH neurons and AMsh glia, respectively, as previously described (Ding et al., 2015; Duan et al., 2020).

### Cell-Specific RNAi

Construction of transgenes for cell specific knock-down requires the fusion of a cell specific promoters to the exon rich regions of the genes. The cell-specific promoter was fused with gene fragments cloned in the sense and antisense orientations, respectively (Chao et al., 2004; Duan et al., 2020). The AMsh glia specific promoter *Pvap-1* was used in this study. The target gene was *egl-19*. The promoters were PCR-amplified from N2 genomic DNA and then recombined with specific donor vector fragments using the In-Fusion PCR Cloning Kit (TaKaRa). The *C. elegans* genes were amplified from genomic DNA, and an exon rich fragment of *egl-19* was amplified with primers as follow:

senseF: ACTTCGACCGCTTCGTCTTG;

senseR: TTTGGAAATGATCGAGCCATTCGGATTGTCCAT

antisenseF: TTGAGGGTACCAAAATTT

antisenseR: ACTTCGACCGCTTCGTCTTGTATC.

These two reactions yield DNA fragments in which the target gene fragment can be transcribed by the cell specific promoter in the sense orientation and in the antisense orientation. Then they are mixed in equimolar amounts and injected, together with a visible selectable marker (*Punc-122::mCherry*), in recipient animals. The fragments for the sense and antisense expression of the target gene were injected at 40 ng^*^μL^−1^ each, together with 30 ng^*^μL^−1^ of co-makers such as *Punc-122::mCherry*. Mock is the control group of the AMsh glia specific *egl-19* RNAi. The mock group animals were injected with the fragments of the sense expression of target geneat 40 ng^*^μL^−1^, together with 30 ng^*^μL^−1^ of co-makers such as *Punc-122::mCherry*.

### Behavioral Assays

Odorant avoidance assays were performed as previously described (Yue et al., 2018; Duan et al., 2020). We placed a 10 μL pipette tip filled with IAA in front of a forward-moving animal, and the response latency for the worm to cease forward movement and initiate reversal (backward movement) was recorded.

Olfactory adaptation assays were performed as we described previously (Duan et al., 2020). Five microliter of undiluted odorant was dropped on the lid of a 3 cm-diameter NGM plate, then D1 adult worms were transferred into the plate and covered the plate with the odorant-containing lid. Sealed the plate with Parafilm. After 5-min adaptation, worms were washed twice with basal buffer [5 mM KPO_4_ [pH 6], 1 mM CaCl_2_, 1 mM MgSO_4_], and then transferred onto normal NGM plates to perform odorant avoidance assays afterwards.

### Calcium Imaging

Animals were immersed in bath solution (145 mM NaCl, 2.5 mM KCl, 1 mM MgCl_2_, 5 mM CaCl_2_, 10 mM HEPES, 20 mM glucose, pH adjusted to 7.3 with NaOH) and subsequently glued on a glass coverslip with a medical grade cyanoacrylate-based glue (Gluture Topical Tissue Adhesive, Abbott Laboratories) (Duan et al., 2020; Cheng et al., 2021, 2022). IAA was first dissolved in DMSO (1:1) as stock solution and then diluted in the bath solution prior to use. The green fluorescent GCaMP5.0 was used to measure the intracellular calcium signals of AMsh glia and ASH neurons (Duan et al., 2020; Cheng et al., 2021, 2022). Fluorescent images were acquired using an Olympus microscope (IX71) under a 40x objective lens coupled with an Andor DL-604M EMCCD camera. Data were collected using the Micro-Manager software. GCaMP5.0 was excited by a ThorLabs blue light (460–480 nm) LED lamp. The fluorescent signals were collected with a 1 Hz sampling rate.

Mechanical stimulation was performed toward the nose tip of the animals through a piezoelectric actuator P-840.30 (Physik Instrumente, Germany) mounted in MPC-325 Micromanipulator (Sutter, USA). The piezoelectric actuator can transform one volt pulse to 4.5 μm mechanical stimulation, triggered by a HEKA EPC-10 amplifier (HEKA, Germany). Odorant stimulation was conducted with IAA (1:100 in bath solution) for 1 min.

To assay the olfactory adaptation of ASH neurons in response to IAA, calcium increases induced by three repetitive applications of IAA (1:100) (30 s-odorant pulses performed with 1-min intervals) were recorded. The baseline calcium level (F_0_) was corrected as the average fluorescence intensity from the last 10 s before each IAA application, and the ratio of the average maximum ΔF/F_0_ changes of peak 2 and peak 3 to the maximum ΔF/F_0_ changes of the first peak was calculated to reflect the adaptation level of ASH neuron.

A detailed glial calcium imaging protocol was described previously.

### Statistical Analysis

Data analysis was performed using GraphPad Prism 6. All data were presented as mean ± SEM. Unpaired two-tailed Student's *t*-test or two-way ANOVA was used to compare data sets. *P* < 0.05 was considered to be statistically significant. Sample sizes were determined by the reproducibility of the experiments and are similar to the sample sizes generally used in the field.

## Data Availability Statement

The original contributions presented in the study are included in the article/Supplementary Materials, further inquiries can be directed to the corresponding author/s.

## Author Contributions

DC, HC, SL YF, DD, and LZ conducted experiments. DC and HC analyzed and interpreted results. DC, HC, UA-S, and LK designed experiments. DC, HC, WZ, and LK wrote the manuscript. All authors contributed to the article and approved the submitted version.

## Funding

This work was supported by grants from the Innovation 2030 Major Project of the Ministry of Science and Technology of China (2021ZD0203300), the National Foundation of Natural Science of China (31771113, 31471023, 31800878, and 31900736), and Zhejiang Provincial Natural Science Foundation (LZ22C090001).

## Conflict of Interest

The authors declare that the research was conducted in the absence of any commercial or financial relationships that could be construed as a potential conflict of interest.

## Publisher's Note

All claims expressed in this article are solely those of the authors and do not necessarily represent those of their affiliated organizations, or those of the publisher, the editors and the reviewers. Any product that may be evaluated in this article, or claim that may be made by its manufacturer, is not guaranteed or endorsed by the publisher.

## Supplementary Material

Supplementary Figure 1 Specific knockdown of *egl-19* in AMsh glia led to moderate change of morphology of AMsh glia but not that of ASH, related to Figure 3. **(A,B)** Morphologies of ASH neurons **(A)** and AMsh glia **(B)** in mock and AMsh glia-specific *egl-19* RNAi worms at D1 stage, respectively. Scale bar, 20 μm. **(C,E)** The lengths of the anterior processes **(C)** and soma **(E)** of ASH neuron in mock and AMsh glia-specific *egl-19* RNAi worms at D1 stage, respectively. Mock (ST2996), *n* = 17; AMsh glia-specific *egl-19* RNAi (ST2995), *n* = 16; unpaired *t*-test. **(D,F)** The ratio of the anterior processes **(D)** and soma **(F)** of ASH neurons to the lengths of the second pharynx in mock and AMsh glia-specific *egl-19* RNAi worms at D1 stage, respectively. The lengths of the second pharynx is calculated as the distance from nose tip to the middle of the second pharynx. Mock (ST2996), *n* = 17; AMsh glia-specific *egl-19* RNAi (ST2995), *n* = 16; unpaired *t*-test. **(G,I)** The lengths of anterior processes **(G)** and soma **(I)** of AMsh glia in mock and AMsh glia-specific *egl-19* RNAi worms at D1 stage, respectively. Mock (ST2674), *n* = 16; AMsh glia-specific *egl-19* RNAi (ST2729), *n* = 27; unpaired *t*-test. **(H,J)** The ratio of the anterior processes **(H)** and soma **(J)** of AMsh glia to the lengths of the second pharynx in mock and AMsh glia-specific *egl-19* RNAi worms at D1 stage, respectively. The lengths of the second pharynx is calculated as the distance from nose tip to the middle of the second pharynx. Mock (ST2674), *n* = 16; AMsh glia-specific *egl-19* RNAi (ST2729), *n* = 27; unpaired *t*-test. Error bars represent SEM. ^***^*p* < 0.001.
