# Supplementary figures and images for: The Voltage-Gated Calcium Channel EGL-19 Acts on Glia to Drive Olfactory Adaptation
Source: Front Mol Neurosci. 2022 Jun 17;15:907064. doi: 10.3389/fnmol.2022.907064 (PMC9247319; doi:10.3389/fnmol.2022.907064)

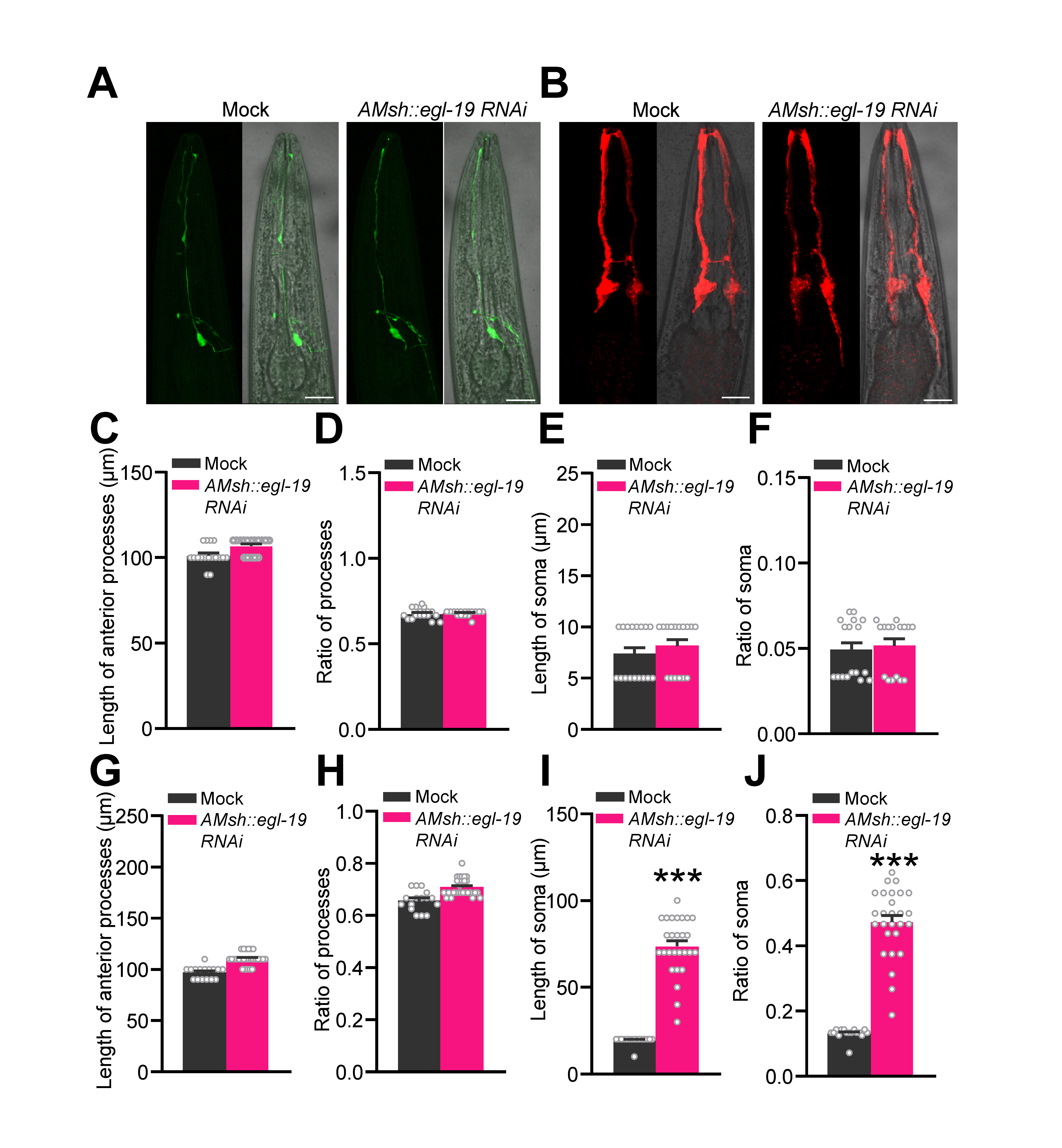

Supplement: Supplementary Figure 1 — Specific knockdown of egl-19 in AMsh glia led to moderate change of morphology of AMsh glia but not that of ASH, related to Figure 3. (A,B) Morphologies of ASH neurons (A) and AMsh glia (B) in mock and AMsh glia-specific egl-19 RNAi worms at D1 stage, respectively. Scale bar, 20 μm. (C,E) The lengths of the anterior processes (C) and soma (E) of ASH neuron in mock and AMsh glia-specific egl-19 RNAi worms at D1 stage, respectively. Mock (ST2996), n = 17; AMsh glia-specific egl-19 RNAi (ST2995), n = 16; unpaired t-test. (D,F) The ratio of the anterior processes (D) and soma (F) of ASH neurons to the lengths of the second pharynx in mock and AMsh glia-specific egl-19 RNAi worms at D1 stage, respectively. The lengths of the second pharynx is calculated as the distance from nose tip to the middle of the second pharynx. Mock (ST2996), n = 17; AMsh glia-specific egl-19 RNAi (ST2995), n = 16; unpaired t-test. (G,I) The lengths of anterior processes (G) and soma (I) of AMsh glia in mock and AMsh glia-specific egl-19 RNAi worms at D1 stage, respectively. Mock (ST2674), n = 16; AMsh glia-specific egl-19 RNAi (ST2729), n = 27; unpaired t-test. (H,J) The ratio of the anterior processes (H) and soma (J) of AMsh glia to the lengths of the second pharynx in mock and AMsh glia-specific egl-19 RNAi worms at D1 stage, respectively. The lengths of the second pharynx is calculated as the distance from nose tip to the middle of the second pharynx. Mock (ST2674), n = 16; AMsh glia-specific egl-19 RNAi (ST2729), n = 27; unpaired t-test. Error bars represent SEM. ***p < 0.001. [file Image_1.JPEG]
